# Phenotypic Characterization of Encephalitis and Immune Response in the Brains of Lambs Experimentally Infected with Spanish Goat Encephalitis Virus

**DOI:** 10.3390/ani10081373

**Published:** 2020-08-07

**Authors:** Ileana Z. Martínez, Claudia Pérez-Martínez, Luis M. Salinas, Ramón A. Juste, Juan F. García Marín, Ana Balseiro

**Affiliations:** 1 Departamento de Sanidad Animal, Facultad de Veterinaria, Universidad de León, 24006 León, Spain; ileanazorhaya.martinez@upaep.mx (I.Z.M.); cperm@unileon.es (C.P.-M.); lsalir00@estudiantes.unileon.es (L.M.S.); jfgarm@unileon.es (J.F.G.M.); 2 Universidad Popular Autónoma del Estado de Puebla, UPAEP Universidad, Puebla 72410, Mexico; 3 Universidad Internacional Antonio de Valdivieso, UNIAV, 47000 Rivas, Nicaragua; 4 Animal Health Department, NEIKER-Instituto Vasco de Investigación y Desarrollo Agrario, 48160 Derio, Bizkaia, Spain; rjuste@neiker.eus; 5 Departamento de Sanidad Animal, Instituto de Ganadería de Montaña, CSIC-Universidad de León, Finca Marzanas, Grulleros, 24346 León, Spain

**Keywords:** Spanish goat encephalitis virus (SGEV), goat, lambs, cell population, immunohistochemistry

## Abstract

**Simple Summary:**

This article studies the local immune response in the central nervous system (CNS) in lambs experimentally infected with Spanish goat encephalitis virus. CNS sections were immunostained to detect microglia, astrocytes, T lymphocytes, and B lymphocytes. In glial foci and perivascular cuffing areas, microglia were the most abundant cell type (45.4% of immunostained cells), followed by T lymphocytes (18.6%) and B lymphocytes (4.4%). Reactive astrogliosis occurred to a greater extent in the lumbosacral spinal cord. Thalamus, hypothalamus, corpus callosum, and medulla oblongata cord contained the largest areas occupied by glial foci. Lesions were more severe in lambs than in goats.

**Abstract:**

Spanish goat encephalitis virus (SGEV), a novel subtype of tick-borne flavivirus closely related to louping ill virus, causes a neurological disease in experimentally infected goats and lambs. Here, the distribution of microglia, T and B lymphocytes, and astrocytes was determined in the encephalon and spinal cord of eight Assaf lambs subcutaneously infected with SGEV. Cells were identified based on immunohistochemical staining against Iba1 (microglia), CD3 (T lymphocytes), CD20 (B lymphocytes), and glial fibrillary acidic protein (astrocytes). In glial foci and perivascular cuffing areas, microglia were the most abundant cell type (45.4% of immunostained cells), followed by T lymphocytes (18.6%) and B lymphocytes (4.4%). Thalamus, hypothalamus, corpus callosum, and medulla oblongata contained the largest areas occupied by glial foci. Reactive astrogliosis occurred to a greater extent in the lumbosacral spinal cord than in other regions of the central nervous system. Lesions were more frequent on the side of the animal experimentally infected with the virus. Lesions were more severe in lambs than in goats, suggesting that lambs may be more susceptible to SGEV, which may be due to species differences or to interindividual differences in the immune response, rather than to differences in the relative proportions of immune cells. Larger studies that monitor natural or experimental infections may help clarify local immune responses to this flavivirus subtype in the central nervous system.

## 1. Introduction

Louping ill is a vector-borne disease endemic of the British Isles and Ireland, which is caused by the louping ill virus (LIV) [1]. In 2011, the Spanish goat encephalitis virus (SGEV), a tick-borne flavivirus subtype closely related to LIV [1], naturally induced non-purulent encephalomyelitis in goats in Spain [2]. SGEV also induced clinical signs (febrile illness and neurological signs such as muscular tremors—mainly located in the neck—ataxia, and/or incoordination) and histopathological lesions in the nervous system in experimentally infected goats and lambs [3,4]. In those studies, lambs developed more severe histological lesions in the central nervous system (CNS) than goats, suggesting greater susceptibility to SGEV [4]. This greater susceptibility has been attributed to species differences and interindividual differences in the immune response; in fact, an effective specific inflammatory response against LIV has been shown to neutralize the spread of the infection in the nervous system in lambs [5].

The pathogenesis of an acute viral infection by flavivirus in the CNS involves complex virus–host interactions in which the recruitment of immune cells into the CNS plays a fundamental role in the outcome of the disease. The response to SGEV in goat CNS has been shown to comprise microglia, T lymphocytes, and, a to lesser extent, B lymphocytes [6]. This is similar to the responses to LIV in mice and lambs [7], to West Nile virus in humans and horses [8,9], to Japanese encephalitis virus in humans [10], and to tick-borne encephalitis virus in humans and non-human primates [11,12]. 

A detailed understanding of the immune response to SGEV in the CNS of different animal species may help clarify the differences among flavivirus. Therefore, the present study examined the phenotype and distribution of microglia, T and B lymphocytes, and astrocytes in the CNS of lambs experimentally infected with SGEV, and the results were compared with the previously reported immune response in goats [6].

## 2. Materials and Methods 

### 2.1. Animals and Sampling

Samples were obtained from eight female 3-month-old Assaf lambs (identified as 14, 18, 19, 21, 25, 26, 28, and 29) from a previous study [4]. In that work, the animals were challenged subcutaneously on the right thorax caudal to the elbow with 1 mL of a suspension containing 1.0 × 10^7^ plaque-forming units per mL of SGEV grown in BHK-21 baby hamster kidney cells. The following postmortem CNS samples were analyzed by histopathology in that study: cerebral cortex, corpus callosum, thalamus, hypothalamus, hippocampus, midbrain, cerebellum, pons, medulla oblongata, and four sections of spinal cord (cervical, thoracic, lumbar, and sacral). Samples were placed in 10% neutral buffered formalin, processed routinely through graded alcohols, and embedded in paraffin wax. The severity of microscopic lesions was rated using our previously reported scale [4]: grade I, only perivascular cuffing; grade II, perivascular cuffing and small foci of glial cells; grade III, moderate non-suppurative encephalomyelitis, perivascular cuffing, diffuse or focal proliferation of glial cells, neuronal degeneration, neuron necrosis, and neuronophagia, demyelination, vacuolation of the neuropil, meningitis, and microvascular changes consisting of reactive endothelium and perivascular edema; and grade IV, which involved the same characteristics as grade III but more severe. In the eight animals, lesions were more frequent and severe in the midbrain, cerebellum, medulla oblongata, and all sections of the spinal cord than in other tissues examined [4]. 

### 2.2. Immunohistochemistry

Serial paraffin-embedded sections (3 µm) were prepared from the CNS samples listed in Section 2.1. and stained with primary antibodies against the following antigens: ionized calcium-binding adaptor molecule 1 (Iba1) to detect microglia cells [9], CD3 to detect T lymphocytes, CD20 to detect B lymphocytes, and glial fibrillary acidic protein (GFAP) to detect astrocytes (Table 1). The sections were de-paraffinized, and endogenous peroxidase activity was blocked by incubating them with 0.5% H_2_O_2_ in distilled water for 30 min. Following antigen retrieval (Table 1), the samples were incubated for 20 min in a humidified chamber with 5% normal serum and 0.1% bovine serum albumin in Tris-buffered saline to prevent non-specific binding. The remaining steps in the protocol and the reagents used throughout immunohistochemistry staining were identical to those described by Martínez et al. (2020) [6]. As a negative control, the slides were subjected to the standard procedure in the absence of the primary antibodies. As a positive control, lamb lymph node tissue was stained against Iba1, CD3, and CD20. As an overall negative control, samples of CNS from healthy lambs were stained with anti-GFAP antibody, and, as positive control, samples from a sheep with CNS degenerative chronic disease were used. 

### 2.3. Evaluation and Quantitation

The stained slides were analyzed under a light microscope (Eclipse E600, Nikon, Japan) equipped with a digital camera (DS-Fi1, Nikon). Cells were counted using NIS-Elements BR imaging software (Nikon). Sections were examined in five randomly selected parenchymal fields at a magnification of 200× [9]. The abundance of microglia, T lymphocytes, and B lymphocytes was expressed as a proportion (%) of all immunolabeled cells within the perivascular cuffing and glial foci. Astrocytes were evaluated using the following semi-quantitative scoring system adapted from a previous study on reactive astrogliosis in human disorders [13]: 1, astrocytes present in healthy CNS tissue, some astrocytes do not express detectable levels of GFAP; 2, mild to moderate reactive astrogliosis; 3, severe diffuse reactive astrogliosis; and 4, severe reactive astrogliosis with compact glial scar formation. 

In order to estimate the total area occupied by glial foci, the areas with glial foci were summed and divided by the total area of the CNS section [6] using Image J software (https://imagej.nih.gov/ij/download.html). To evaluate the spatial distribution of glial foci, we chose medulla oblongata sections because they were consistently affected and had a similar size in all lambs examined [4] and we studied them using light microscopy (BX51, Olympus, Japan) and the imaging software OlyVIA 2.91 (Olympus, Japan). The images were analyzed using VS-ASW 2.8 software (Olympus, Japan). 

### 2.4. Statistical Analysis 

**Figure 1:**
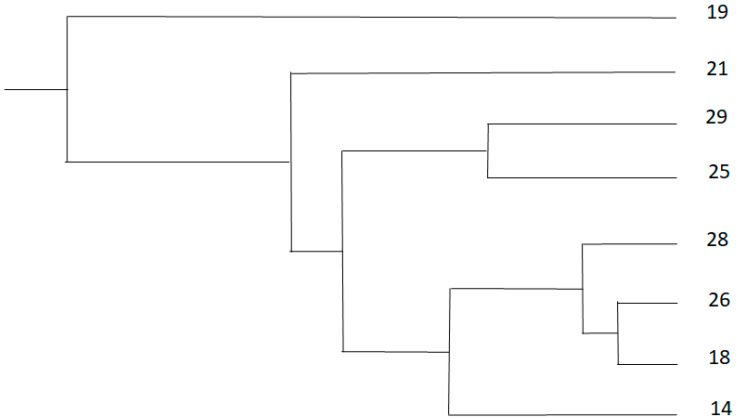
Cluster analysis of lambs experimentally infected with Spanish goat encephalitis virus (SGEV) in terms of lesion severity using the centroid method in the SAS CLUSTER procedure. Lambs 19, 21, 25, and 29 were assigned to cluster A (mild to moderate lesions), while lambs 14, 18, 26, and 28 were assigned to cluster B (moderate to severe lesions).

The proportions of immunolabeled cells that were microglia or T or B lymphocytes were submitted to an arcsin square-root transformation in order to meet the normality criterion for statistical analyses. Then, the results were subjected to cluster analysis by the centroid method in the SAS CLUSTER procedure (SAS, Cary, NC, USA), which generated two clusters: cluster A comprised lambs 19, 21, 25, and 29, which showed a relatively loose relationship; and cluster B comprised lambs 14, 18, 26, and 28, which showed a relatively tight relationship. Microscopic lesions in each cluster did not fit nearly within the four-grade scoring system: cluster A lesions were mild to moderate (grades I and III), while cluster B lesions were moderate to severe (Figure 1) (grades III and IV) [4]. These clusters were considered to better represent the severity of lesions in the study sample of lambs. As a consequence, “cluster” was added as a variable in order to capture population variability potentially more accurately than only with individual values. 

A general linear model was constructed using the GLM procedure in SAS with the following dependent variables: arcsin-transformed proportions of microglia, T lymphocytes, and B lymphocytes; log-transformed semi-quantitative scores for astrocytes; and proportion of total stained area that was positive for Iba1. The independent variables were: CNS region, type of lesion (perivascular cuffing or glial foci), cluster, and their first-order interactions. 

Pairwise least-square means comparisons were carried out using Student’s t test as implemented in the LSMEANS statement in SAS. Statistical significance was accepted at *p* < 0.05, while *p* = 0.05–0.1 was taken to indicate a tendency [6]. 

Potential differences in the number of lesions (glial foci) between the infected side of the animal and the opposite side (laterality effects) were examined using the chi-squared test in the FREQ procedure in SAS and confirmed using the log-transformed foci counts submitted to analysis of variance of the generalized linear model. Laterality effects were also confirmed using a least-squares mean Student’s t test with the LSMEANS statement in a model with three independent variables: cluster, side, and their interaction [6]. 

### 2.5. Ethics Approval

Sampling procedures and SGEV challenge in the previous study by Salinas et al. (2017) [4] were approved by the Animal Research Ethics Committee of the Community of Junta de Castilla y León, Spain (reference number ULE_010_2015). Experiments were conducted in accordance with Spanish and European legal requirements and guidelines on animal experimentation and welfare. 

## 3. Results

### 3.1. Microglia 

**Figure 2:**
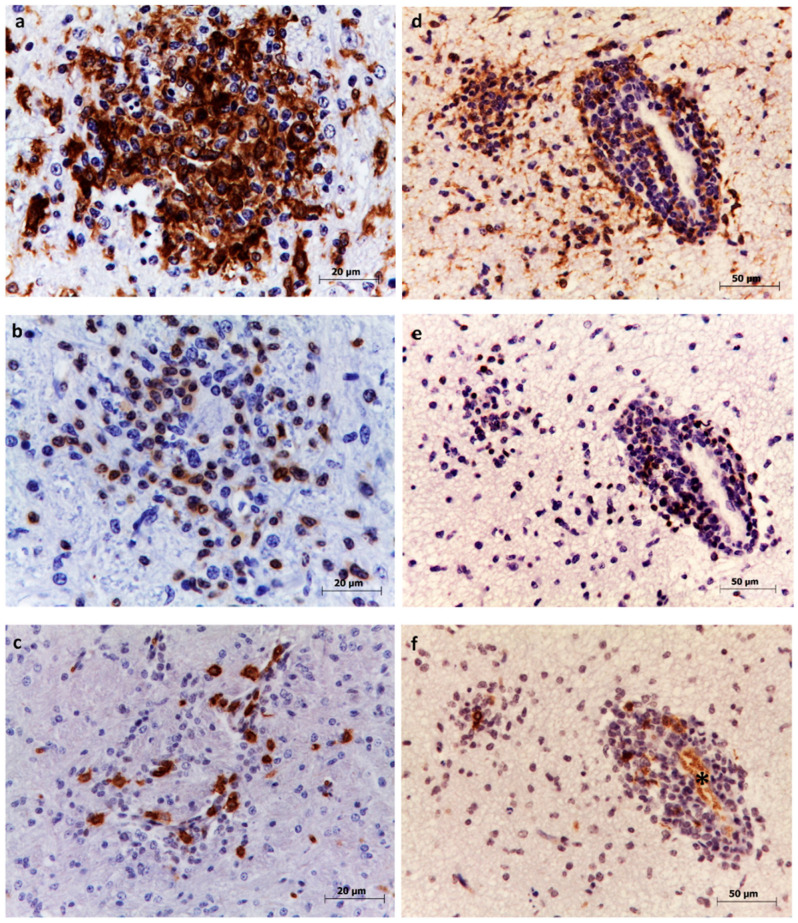
Immunohistochemistry to detect microglia, T lymphocytes, and B lymphocytes in thalamus (**a**–**c**) and hippocampus (**d**–**f**). Microglia were identified based on labeling with anti-Iba1 antibodies. Abundant cells are visible within glial foci (**a**) and in the perivascular infiltrate (**d**). T lymphocytes were identified based on labeling with anti-CD3 antibodies. Moderate numbers are visible in the glial focus (**b**) and in the perivascular cuff (**e**). B lymphocytes were identified based on labeling with anti-CD20 antibodies. Few numbers are visible within the glial focus (**c**) and perivascular infiltrate (**f**). Erythrocytes were also immunolabelled (asterisk) but were not included in the counting.

Microglia was the most abundant cellular type (Figure 2a,d), accounting for an average of 45.4% of the three immunostained cell populations. Based on lesion severity, lambs were classified into cluster A with mild to moderate lesions and cluster B with moderate to severe lesions (Section 2.4). Microglia was significantly more abundant in cluster B (59.3%) than in cluster A (31.5%, *p* < 0.0001; Table 2). Microglia was also significantly more abundant in cortex (55.3%) and hippocampus (55.2%) than in cervical spinal cord (34.4%) or lumbosacral spinal cord (31.6%; both *p* < 0.0001). Additionally, they were significantly more abundant in glial foci (55.2%) than in perivascular cuffing (35.5%, *p* < 0.0001). 

Glial foci were significantly more numerous in cluster B, where they covered a significantly larger proportion of affected areas (15.9%) than in cluster A (8.4%, *p* < 0.0001). Glial foci were significantly more numerous in thalamus, hypothalamus, corpus callosum, and medulla oblongata (19.5% of affected areas) than in cervical spinal cord (5.6%, *p* = 0.0682) and lumbosacral spinal cord (6%, *p* = 0.0522). 

### 3.2. T lymphocytes

T lymphocytes were the second most abundant cell type (Figure 2b,e), accounting for 18.6% of the three immunostained cell populations. Like microglia, they were significantly more abundant in cluster B (24.8%) than in cluster A (12.5%; *p* < 0.0001; Table 2). Their proportion was higher in pons and cerebellum (25.1%) than in cervical spinal cord (11.6%, *p* = 0.0139) and was not significantly different between perivascular cuffing (20.6%) and glial foci (16.6%, *p* = 0.8350). 

### 3.3. B lymphocytes

B lymphocytes were the less abundant cell type (4.4%) (Figure 2c,f), and their proportion was similar in cluster B (6.4%) and cluster A (2.4%, *p* = 0.1213; Table 2). All CNS regions contained comparable proportions of B lymphocytes, ranging from 3.4% in cortex and medulla oblongata to 5.8% in midbrain. Their proportions were slightly higher but not significant in perivascular cuffing (6.3%) than in glial foci (2.5%, *p* = 0.8659). 

### 3.4. Proportions of Microglia, T lymphocyte,s and B Lymphocytes According to Lesion Location and Severity

The proportions of microglia were higher than those of T and B lymphocytes in all CNS regions (Table 2), and the differences were significant in the cortex (microglia vs. B lymphocytes, *p* < 0.0001; microglia vs. T lymphocytes, *p* = 0.0051), thalamus, hypothalamus, and corpus callosum (microglia vs. B lymphocytes, *p* < 0.0001), hippocampus (microglia vs. B lymphocytes, *p* < 0. 0001; microglia vs. T lymphocytes, *p* = 0.0051), midbrain (microglia vs. B lymphocytes, *p* = 0.0047), pons and cerebellum (microglia vs. B lymphocytes, *p* = 0.0002), and medulla oblongata (microglia vs. B lymphocytes, *p* = 0.0001). Proportions of T and B lymphocytes were not significantly different in any region. 

The proportion of microglia cells was higher than those of both lymphocyte types in cluster B (both *p* < 0.0001), and the proportion of T lymphocytes was higher than that of B lymphocytes (*p* < 0.0001). Similar results were observed in cluster A. The proportions of microglia were not significantly different between clusters A and B (31.5% vs. 59.3%; *p* = 0.1213), but the proportions of lymphocytes were significantly higher in cluster B (T, 24.8% vs. 12.5%, *p* < 0.0001; B, 6.4% vs. 2.4%, *p* < 0.0001). 

### 3.5. Astrocytes

**Figure 3:**
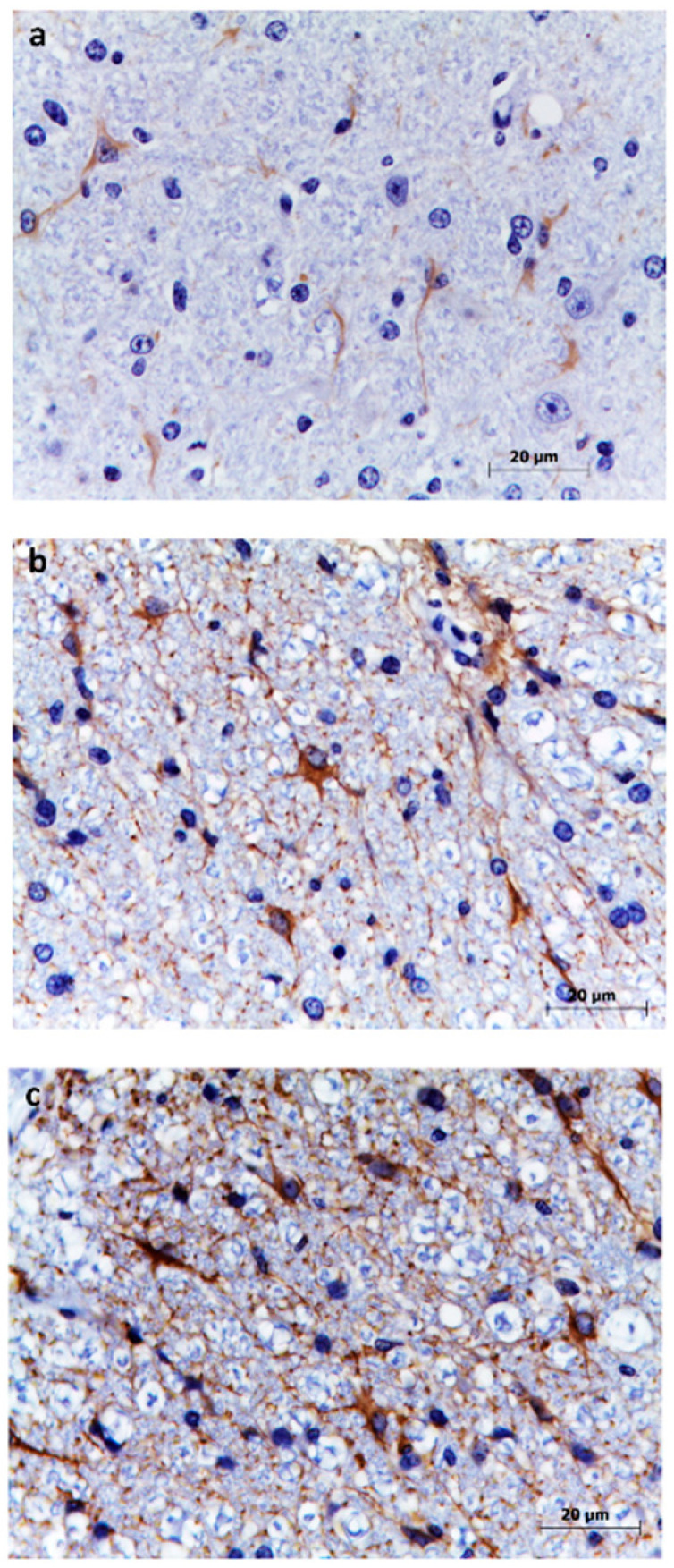
Grading of reactive astrogliosis in cervical spinal cord (**a**–**c**) using antibodies against GFAP. (**a**) Representative micrograph showing grade 1 (low) reactive astrogliosis. (**b**) Representative micrograph showing grade 2 (mild to moderate) reactive astrogliosis. (**c**) Representative micrograph showing grade 3 (severe) reactive astrogliosis.

Astrocytes were scored on a four-point scale (Figure 3), and we observed no cases with score 4. The frequencies of astrocyte scores did not differ significantly between regions or clusters. The mean astrocyte score was the highest in the lumbosacral spinal cord (2.9%), decreased in the cervical spinal cord and medulla oblongata (2.5%), and was the lowest in the hippocampus (2%, Table 2). 

### 3.6. Lesion Count and Laterality 

**Figure 4:**
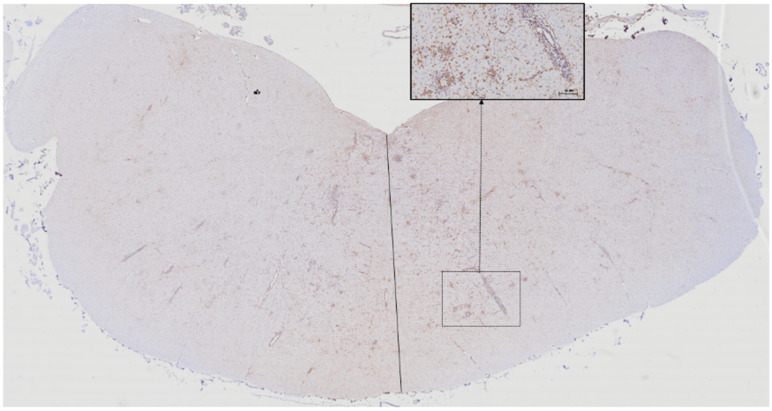
Complete cross-section of the medulla oblongata immunostained for microglia, showing numerous glial foci on the same side of the tissue (right side) as virus injection. Inset: detail of the affected parenchyma.

Lesions were marginally more frequent on the right side of the CNS (62.3%) (Figure 4), the side of virus injection, than on the left side (37.7%, *p* = 0.0450), although the total mean lesion count did not significantly differ between the right and the left sides (11.9 vs. 6.79, *p* = 0.3242). Similarly, differences in lesion numbers between the right and the left sides were not significant in cluster A (1.3 vs. 0.0, *p* = 0.2886) or cluster B (4.6 vs. 2.0, *p* = 0.224).

## 4. Discussion

This study characterized and quantified the distribution of immune cells in the CNS of lambs experimentally infected with SGEV. The inflammatory cells were predominantly microglia, with a moderate number of T lymphocytes and a smaller number of B lymphocytes. These findings are similar to those of previous studies on SGEV in goats [6] and on LIV and other flaviviruses in horses, humans, and non-human primates [7,8,9,10,11,12,14,15]. Microglia, which are of mesodermal origin, play a key role in the innate and adaptive immune responses in the CNS [10]; in fact, they are the first cells that respond to CNS infection [12,16]. Infection itself and the resulting neuronal damage activate the recovery in microglia of their amoeboid properties such as motility and of their macrophagic function [10,12,16]. This activation increases neuronal cell death, astrogliosis, and production of pro-inflammatory cytokines [10,12,16]. 

The proportion of microglia was higher in lambs in the present study than in goats in previous work [6], which may reflect the greater susceptibility of lambs to tissue damage [17]. In this regard, 5 out of 9 and 2 out of 9 lambs showed type III and type IV microscopic lesions, respectively, whilst only 4 out of 9 goats showed grade III lesions, and none grade IV lesions with an identical challenge [3,4]. Moreover, in the present study the proportion of microglia was higher in cluster B and was related to more extensive tissue damage. In studies of human and animal infection with Japanese encephalitis virus, microglia predominated in the thalamus and hippocampus, the primary affected brain regions [18]. We also observed the greatest relative abundance of microglia in thalamus and hippocampus, although these regions were not the most seriously affected by SGEV [4]. 

T lymphocytes were the second most abundant cell population, as reported in flavivirus infections in goats, lambs, humans, non-human primates, horses, and mice [6,7,9,11,15,17,19]. T lymphocytes control viral infections in the CNS by destroying infected cells, producing cytokines, stimulating phagocytic activity of microglia, and stimulating local antibody production by B lymphocytes [12,20]. High numbers of T lymphocytes have been linked to fatal outcome in human patients infected with tick-borne encephalitis virus [21]. T lymphocytes were more abundant than B lymphocytes, but the difference was not significant, similar to the case in goats infected with SGEV [6] and in lambs infected with LIV [22]. A higher abundance of T lymphocytes than of B lymphocytes has been associated with acute disease in horses infected with West Nile virus [9]. 

B lymphocytes were the less numerous cellular type and were more abundant in perivascular spaces (6.3% of all immunostained cells) than in parenchyma (2.5%), similar to what has been reported in goats, humans, and non-human primates infected with flavivirus [6,11,12,17,23]. This might reflect antibody-mediated tissue damage in lambs [17]. The proportion of B lymphocytes was lower in lambs in the present study than in goats in previous work [6], which may reflect a lower specific humoral cell-mediated response in lambs. 

Reactive astrogliosis was observed in the CNS of mice, lambs, goats, humans, and mice infected with flaviruses [6,23,24,25], especially in persistent infections [25]. In our study of lambs, most CNS sections received an astrogliosis score of 2, characteristic of diffuse innate immune activation in response to viral infection [13]. An astrogliosis score of 3 was observed mainly in the spinal cord near the pia mater, which is the most likely entry route for the virus into the CNS, where astrocytes are in the lining of brain capillaries and pia mater and are the first cells to come in contact with the virus [25]. GFAP expression has been associated with the greatest damage in humans infected with West Nile virus [23]; this is similar to the present study’s finding of a particularly strong GFAP immunostaining in spinal cord and medulla oblongata, where histological lesions following SGEV infection are more frequent and severe [4].

In our SGEV-infected lambs, glial foci were significantly more abundant in thalamus, hypothalamus, corpus callosum, and medulla oblongata; SGEV-infected goats, in contrast, showed more foci in medulla oblongata and spinal cord [6]. Therefore, immunohistochemistry can help identify tissues preferentially affected by different flaviviruses, otherwise difficult to detect using conventional hematoxylin-and-eosin staining, as already demonstrated for tick-borne encephalitis and Japanese encephalitis [14,16]. We observed lesion predominance on the same side of the medulla oblongata as that of the subcutaneous virus injection, similar to results obtained in goats [6]. This preference supports a neurotropic route of virus movement [4].

## 5. Conclusions

The immune response to SGEV in lambs appears to involve a combination of microglia and T lymphocytes, suggesting that B lymphocytes proliferate during infection. The proportion of microglia among immune cells was higher in SGEV-infected lambs than in goats, which may reflect the greater lesion severity in sheep. Nevertheless, the relative proportions of four types of cells (microglia, T and B lymphocytes, and astrocytes) in the CNS were similar between infected lambs and goats. Therefore, we may conclude that lambs might be more susceptible to SGEV due to species differences or to different immune responses between individuals, rather than to differences in the relative proportion of immune cells. Larger studies that analyze flavivirus infections over longer periods may further increase our understanding of the immune response.

## Figures and Tables

**Table 1:** Immunohistochemical protocols used for cellular type characterization.

Antibody/Specificity	Clone n°	Isotype	Manufacturer	Antigen Retrieval	Dilution
Iba1/Macrophages/microgial cells	Polyclonal 019-19741	Rabbit IgG	FLUJIFILM-Wako Chemicals Europe GmbH, Neuss, Germany	Citrate pH 6.0 in microwave in 20 min	1:1000
CD3/T lymphocytes	Monoclonal NCL-L-CD3-565	Mouse IgG	Novocastra, Leica Biosystem, Neucastle, United Kingdom	Citrate pH 6.0 in microwave in 20 min	1:500
CD20/B lymphocytes	Polyclonal PA5-16701	Rabbit IgG	ThermoFisher, Massachusetts, USA.	Citrate pH 6.0 in steamer for 20 min	1:200
GFAP/Astrocytes	Monoclonal MCA-5C10	Mouse IgG	EncorBiotechnology, Gainesville, Florida, USA	Citrate pH 6.0 in microwave in 20 min	1:8000

GFAP, glial fibrillary acidic protein; Iba1, ionized calcium binding adaptor molecule 1.

**Table 2:** Proportion (%) of all immunostained cells, i.e., microglia, T or B lymphocytes, or astrocytes, in lambs experimentally infected with SGEV, stratified according to lesion severity, central nervous system (CNS) region, and lesion type.

Variable	Level	Microglia (Iba1)		T lymphocytes (CD3)		B lymphocytes (CD20)		Astrocytes (GFAP)	
		Mean	SE	Mean	SE	Mean	SE	Mean	SE
Cluster	A (mild to moderate lesions)	31.5	2.3	12.5	2.3	2.4	2.3	2.4	1.1
	B (moderate to severe lesion)	59.3	2.3	24.8	2.34	6.4	2.34	2.3	1.1
Region	Cortex	55.3	4.7	16.4	4.7	3.4	4.70	2.1	2.2
	Thalamus, hypothalamus, corpus callosum	51.4	4.7	18.9	4.7	4.8	4.7	2.4	2.2
	Hippocampus	55.2	4.7	16.0	4.7	3.9	4.7	2.0	2.2
	Midbrain	42.1	4.7	21.6	4.7	5.8	4.7	2.1	2.2
	Pons, cerebellum	45.8	4.7	25.1	4.7	4.5	4.7	2.3	2.2
	Medulla oblongata	47.6	4.7	24.7	4.7	3.4	4.7	2.5	2.2
	Cervical spinal cord	34.4	4.7	11.6	4.7	5.6	4.7	2.5	2.2
	Lumbosacral spinal cord	31.6	4.7	14.8	4.7	3.8	4.7	2.9	2.2
Type of lesion	Glial foci	55.3	2.3	16.6	2.3	2.5	2.3	2.3	0.1
	Perivascular Cuffing	35.5	2.3	20.6	2.3	6.3	2.3	-	-

SE, standard error. *Between clusters:* Proportions of microglia and T lymphocytes were significantly different (*p* < 0.0001), whereas proportions of B lymphocytes (*p* = 0.1213) and astrocytes (*p* = 0.3435) were not. *Among CNS regions*: Proportions of microglia in cortex and hippocampus were different from proportions in cervical and lumbosacral spinal cord (*p* < 0.0001). Proportions of T lymphocytes in pons and cerebellum differed from those in cervical spinal cord (*p* = 0.0139). Proportions of B lymphocytes did not differ significantly among regions (*p* = 0.5056), nor did proportions of astrocytes (*p* = 0.2124). *Between type of lesion*: Proportions of microglia differed (*p* < 0.0001), whereas proportions of T lymphocytes (*p* = 0.8350) or B lymphocytes (*p* = 0.8659) did not.
